# Severity of error in hierarchical datasets

**DOI:** 10.1038/s41598-023-49185-z

**Published:** 2023-12-11

**Authors:** Satwik Srivastava, Deepak Mishra

**Affiliations:** 1 Department of Mathematics, Indian Institute of Technology Jodhpur, Jodhpur, India; 2 Department of Computer Science and Engineering, Indian Institute of Technology Jodhpur, Jodhpur, India

**Keywords:** Computer science, Image processing, Machine learning

## Abstract

Classification tasks today, especially for the medical domain, use datasets which are often hierarchical. These tasks are approached using methods that consider the class taxonomy for predicting a label. The classifiers are gradually becoming increasingly accurate over the complex datasets. While increasing accuracy is a good way to judge a model, in high-risk applications, it needs to be ensured that even if the model makes a mistake, it does not bear a severe consequence. This work explores the concept of severity of an error and extends it to the medical domain. Further, it aims to point out that accuracy or AUROC alone are not sufficient metrics to decide the performance of a model in a setting where a misclassification will incur a severe cost. Various approaches to reduce severity for classification models are compared and evaluated in this work, which indicate that while many of them might be suited for a traditional image classification setting, there is a need for techniques tailored toward tasks and settings of medical domain to push artificial intelligence in healthcare to a deployable state.

## Introduction

**Figure 1:**
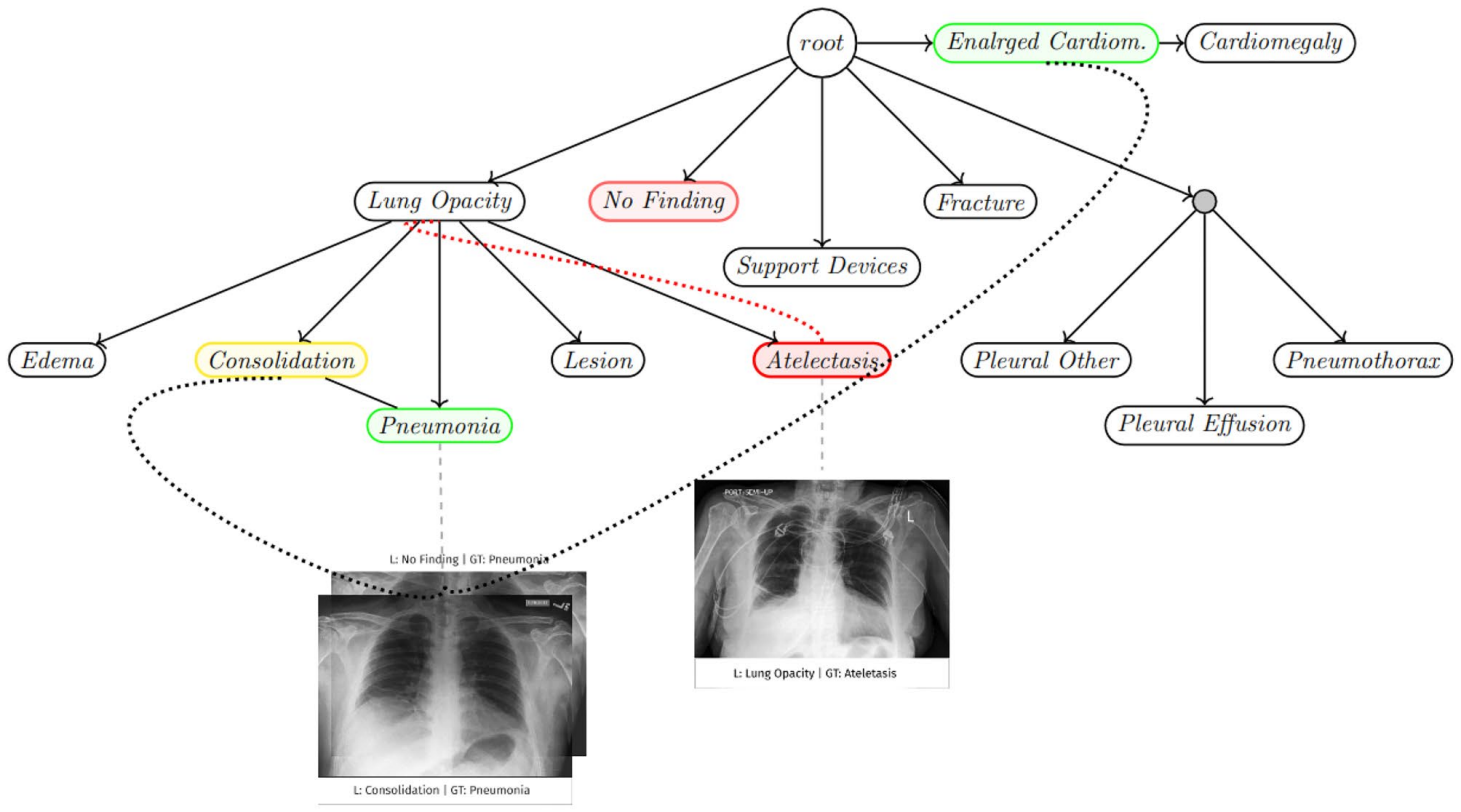
Visualizing severity through the CheXpert hierarchy tree. When the model predicts *Atelectasis* as *Lung Opacity*, the severity value is low as the two diseases are close in the hierarchy tree. Hence, the cost of misdiagnosis is relatively low. Similarly, for a sample where *Pneumonia* is predicted as *Consolidation*, we have a low severity value. On the other hand, *Pneumonia* being predicted as *No Finding*, the cost of the error made is much higher due to both nodes having only the root node as a common ancestor.

With onset of deep learning in the field of image analysis and computer vision, many frameworks have been proposed that solve various image analysis-based problems such as image classification, labelling, segmentation, etc. Each new advancement in the field targets better accuracy scores of the models. Specially the progress in medical image analysis and classification have led to a near-deployment level finesse of AI in healthcare^1^. Secinaro et al.^2^ point out the areas of healthcare where deploying an AI solution is a viable outlook. Several studies have also explored the particular areas of applications. Davenport and Kalakota^3^ note, that while AI is not on track to replace humans in this field, it can certainly help augment the work of experts to provide a better service in such sectors. To that end, several approaches have been proposed for various tasks in healthcare. One of the most common applications of AI in healthcare is augmenting the tasks such as classification, detection and labelling. Several different frameworks and approaches showing high performance have been reported over datasets of predominantly medical images obtained through X-Rays or other modalities such as MRIs and CT Scans^4–7^. These standard datasets like CheXpert, Mimic-III, and Padchest^8–10^ have the labels arranged in a hierarchy (Fig. 1). The abundance of hierarchical datasets and the inherent hierarchy that can be derived from various datasets is discussed further in “Background”.

The task in which deep learning has achieved a remarkable success is the automated diagnosis driven by image classification. In general, classification approaches are judged based on their accuracy or AUROC scores. Accuracy in simple terms is defined by Eq. (1) for a standard binary classification problem.

1 $$\begin{aligned} \hbox {Accuracy} = \frac{TP + TN}{TP + TN + FP + FN} \end{aligned}$$

where $TP,\, TN,\, FP,\, FN$ are the number of True Positive, True Negative, False Positive and False Negative observations. Similarly, area under the reciever operating statistics curve (AUROC) gives the performance measurement for the classification problems at various threshold settings and is defined by Eq. (2).

2 $$\begin{aligned} \hbox {AUROC} = \frac{TPR}{FPR} \end{aligned}$$

Here, *TPR* and *FPR* are the *True Positivity Rate* and the *False Positivity Rate*. It tells how much the model is capable of distinguishing between classes. Higher the AUROC, better the model predicts a class as its actual label or ground truth value. While these are robust measures of judging the performance of such deep learning networks and thus are essential to keep track of, they do not indicate the nature of errors themselves. In a setting where each error or misdiagnosis can lead to a high cost, these errors need to be ranked, and it should be ensured that the model does not always populate the misdiagnosis with high-impact errors. To understand this better, consider the case study by Neale et al.^11^, which analyses how misdiagnosis can occur in various phases of the diagnostic process and how costly it is at each stage. The study points out that system diagnostic failures can be a major cause of misdiagnosis in 25–$65\%$ of the total assessed cases. Furthermore, the case study by Braun et al.^12^ sheds light on the diagnostic errors that occur in various modes of analysis like Chest X-rays, lab reports and other modes of medical information analysis. Thus in the field of healthcare, misdiagnosis is a significant issue, and different types of misdiagnosis cannot be clubbed together. Therefore, errors made by the networks should not carry the same weight. However, most classification tasks treat each error equally i.e. the impact of each misclassification by the model is equal.

For example, a network which uses X-ray images to predict the type of disease or condition a person has will often be judged by its ability to predict each class accurately, and the model will consider one error equally as wrong as any other error. Consider a case of effusion and consolidation. These are diseases where fluid fills up in or around the lungs. Thus, the feature maps extracted from their samples might appear similar to a regular deep feature extraction model. However, a misdiagnosis between these can lead to vastly different treatments for the diseases and lead to potential harm to the patient. Similar issues are highlighted by Oh et al.^13^ in their work. In such cases, each error cannot be weighted equally. Thus, a model must learn to avoid mistakes that have severe consequences.

The solution to this problem requires a parameter that provides a way to measure how critical an error can be. A potential term for this is *Severity of Error* (SoE) which can be used to quantify an error’s importance when it is made.

This work aims to explore SoE and its use in the medical domain, further evaluating it on hierarchical datasets presently used by State-of-The-Art (SoTA) methods for AI in healthcare. Additionally, different proposed frameworks are explored which aim to reduce severity. The experiments performed using these methods, thus, aim to show the importance of SoE for the classification tasks with high-risk potency.

The main contributions of this work are the following:

- The concept of Severity of Error (SoE) is introduced for the multi-label classification framework in the medical domain increasing its generalizability and adaption in various different use cases.
- Introduction of a novel way of calculating the Severity of Error for classification tasks.
- A modified CRM Framework is introduced to make it adaptable for the multi-label classification tasks as well as more generalizable for the classification task.
- This work lays down the foundation for error severity of deep learning models in medical domain and opens up a new direction for future works.

## Background

There is a growing concern over the prediction of unsafe labels in the task of medical image analysis^14–17^. As highlighted in the above, we need parameters, such as SoE, to quantify the degree of harm an incorrect prediction can cause. Intuitively, SoE is a quantitative measure of how costly an error made by the model in any particular setting is. This cost can be calculated using various methods. The principle of Cost-Sensitive Learning^18^ uses a predefined cost matrix *C* where each entry $c_{i, j}$ reflects the cost (or penalty) that will be incurred when our model predicts the *i*th class where the ground truth is $j \, (\forall \, i \ne j)$. However, this is largely based on an experimental approach that can not be easily automated without the presence of a domain expert. The notion that an error has a higher cost than the other, intuitively implies that each label is related to the other through a taxonomic relationship. Thus the datasets explored in this work are all hierarchical.

To this end, it is observed that two major representations of the class hierarchy occur in the standard medical and non-medical datasets. The first one is represented by a hierarchy tree where the leaf nodes represent the class labels of the dataset. These leaf nodes might not be at the same level in the hierarchy tree; however any internal node (nodes with a children) is treated as a superclass and hence does not occur in the prediction. We here refer to this type of hierarchy as *type-1* hierarchy which is seen in datasets like tieredImageNet^19^, and iNaturalist^20^. However, in medical domain another kind of hierarchical organisation of data where each node is a class label is more common. Here every node, which may or may not include the root node, is used as a label for classification. This is referred as *type-2* hierarchy. Some examples of medical datasets with *type-2* hierarchy include CheXpert, MIMIC and FractureNet data^8, 9, 21^.

Despite the difference in hierarchical organisation, the dissimilarity between two nodes of a hierarchical datasets, i.e. the measure of how “far” a particular predicted label is from the ground truth value, can be extracted from the class hierarchy using a common metric such as Lowest Common Ancestor (LCA) distance between the two nodes^22, 23^. This is represented by Eq. (3).

3 $$\begin{aligned} {\mathcal {S}}_i = \textsc {lca}({\hat{y}}_i, y_i) \end{aligned}$$

here ${{\mathcal {S}}_i}$ is the severity of the *i*th sample, ${{\hat{y}}_i}$ is the *predicted value*, and ${y_i}$ is the *ground-truth*.

**Figure 2:**
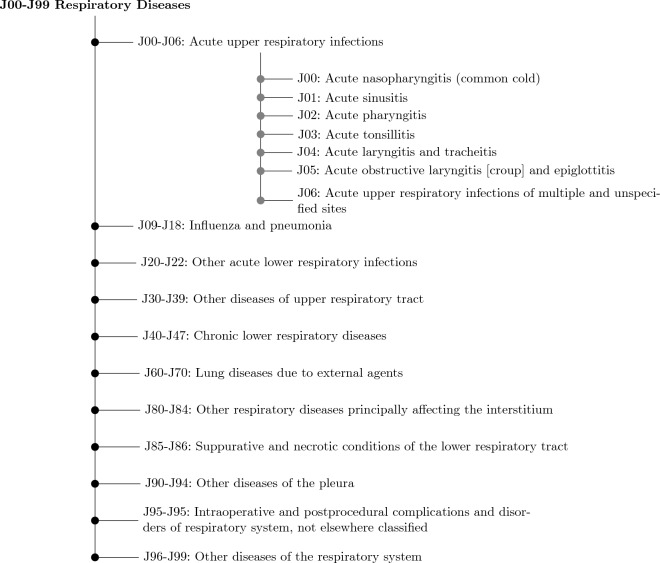
The ICD-10 codes^24^. Each type of diagnosis for a condition has an ICD-10 code used internationally in any form of medical image analysis. Each scan is packaged in the DICOM format, which includes such a code. These codes are hierarchically arranged and thus the hierarchy in any medical imaging dataset can be easily extracted and built.

Figure 1 visualizes the severity of various sample predictions. We can see a high severity when a model predicts *Atelectasis* as *Enlarged Cardiomegastinium*. Similarly, for a sample where *Pneumonia* is predicted as *No Finding*, we have a high severity value. On the other hand in case of *Pneumonia* being predicted as *Consolidation* the cost of the error made is comparatively less as both nodes coming under the same branch. This can further be studied as the inbuilt hierarchy in medical data can be found and built upon using ICD-9 and ICD-10 codes which often groups similar conditions under a common related root or super-class. Figure 2 shows the inbuilt hierarchy of respiratory diseases and ailments, which can be built from their ICD-10 codes. Such a structure can be exploited in the classification of medical data and severity analysis. In the tree, we can see that each leaf is a disease, and its parent is the class of that disease. Similar to the label *J* constituting Respiratory diseases, such a taxonomy can be built for all other diseases using other labels. This indicates that there is a deep and rich hierarchy present in all the medical data.

The SoE in the current analysis is closely related to the class hierarchy. However, leveraging class hierarchies to improve classifiers is not new. Many authors have incorporated it in their works where hierarchical datasets are used to obtain better classification results^25^. For example, a hierarchical loss can integrate the class hierarchy into the training objective. Thus any model that learns through such loss function should be able to produce less severe results. Dimitrovski et al.^26^, in their work on the IRMA dataset, use a *Hierarchical Error Measurement*^27^ metric proposed in the dataset itself. It assigns a higher value to an error that occurs at a higher level of the predictive clustering tree (PCT)^8^. This is a case of a Hierarchical classification clubbed with a Hierarchical Error. Bertinetto et al.^28^ proposed Hierarchical Cross Entropy which incorporates the class hierarchy of the labels while performing error calculations. In contrast, Karthik et al. used a post-inference framework of conditional risk minimization (CRM)^23^ in their work to leverage class hierarchies to reduce the top-k error bound of their models.

As we move towards the application of AI in more and more domains, the risk of such errors is an important consideration. Moreover, with the increase of hierarchical datasets in the medical domain^8–10, 21, 29, 30^, SoE can be used for evaluation before the deployment of any new model for any such tasks. However, all the experiments that have been previously done focus on classification tasks on popular datasets of a general classification domain like the iNaturalist dataset^20^ or the tieredImageNet dataset^19^. This work focuses on the medical domain and observes how introducing severity as a parameter changes the deep learning model performances in high-risk classification. Further, it analyses the effectiveness of the current methods in this domain and the scope of further improvement.

## Method

There are three possible ways to incorporate severity in a classification task. These include tuning the dataset for severity, i.e. introducing a class taxonomy in it or balancing out the data samples and creating a cost matrix for the labels, which is termed a pre-training modification, while severity can also be injected into the training process and further it can also be used at inference time to reduce the cost of errors.

1. The concept of dataset preprocessing and cost sensitive learning^18^ requires that the dataset be organized into a hierarchy tree and a cost matrix (*C*) be formed where each element $c_{ij}$ of the matrix denotes the cost between the *i*th and the *j*th label. This cost matrix can then be included in different phases of the model learning to batch sampling pipeline. While this process is easy to do these may be inaccurate as cost matrix definition can have multiple different techniques.
2. Incorporating SoE in the training phase itself through the use of custom loss functions^28^ such that any model can learn to choose the labels with the least severity. This method is very costly as the model will have to be trained on the specific loss function which would penalize inaccuracy as well as high severity however this method can be used to incorporate severity into any general model.
3. Using a post inference technique^23^ to lower severity for the model at inference time such that the model can select the label reordered according to the technique. While this method is fast the model itself will not learn anything here and thus this method becomes hyper-local in its deployability.

An error is a top-1 prediction of a classifier that differs from the ground truth. Thus, qualitatively SoE is an indirect measure of the impact a top-1 error/mistake has (as real-world consequences) if taken at face value and utilized without any form of correction.

This work explores two possible methods of reducing the severity of a classifier. First, using the hierarchical cross entropy loss (HXELoss) function for classifier training, which can incorporate the information about the hierarchy of a dataset directly into the classifier^28^. Second, using the CRM framework^23^ to populate a pool of top-k results with the least severity samples at the time of inference.

### Hierarchical cross entropy

This method comes under the umbrella of using hierarchical loss functions which are the loss functions that incorporate class hierarchy such that a more distant prediction (from the ground truth) automatically has a higher value of the loss.

As described in^27^, the hierarchy ${\mathcal {H}}$ present in a dataset is a categorical distribution over the levels of the taxonomic hierarchy tree. This is defined in terms of conditional probabilities over classes and is denoted as $p(C^{(l)})$ where (*l*) is the level of the hierarchy tree at which this class or node is present. Thus root ${\mathbb {R}}$ can be denoted as $C^{(0)}$. Now a path connecting a leaf node *C* to the root can be denoted as $C = C^{(0)}, \ldots C^{(h)} = {\mathbb {R}}$. Thus *p*(*C*) can be factorized as:

4 $$\begin{aligned} p(C) = \prod _{l=0}^{h-1} p(C^{(l)} | C^{(l+1)}) \end{aligned}$$

here $h == h(C)$ is the height of the node *C*. The above conditional can be conversely written as follows:

5 $$\begin{aligned} p(C^{(l)} | C^{(l+1)}) = \frac{\sum _{A \in \hbox {Leaves}(C^{(l)})} p(A) }{\sum _{B \in \hbox {Leaves}(C^{(l+1)})} p(B)} \end{aligned}$$

this leads to hierarchical cross entropy (HXE) proposed by Bertinetto et al.^28^ where the hierarchical loss function is defined as:

6 $$\begin{aligned} {\mathcal {L}}_{\hbox {HXE}}(p, C) = - \sum _{l=0}^{h-1} \lambda (C^{(l)}) \log p(C^{(l)} | C^{(l+1)}) \end{aligned}$$

here $\lambda (C^{(l)})$ is the weight associated with the edge connecting the node $C{(l+1)}$ to $C^{(l)}$.

### The conditional risk minimization framework

Another way to incorporate severity in the classification task is to include it in the post-validation/inference phase. As shown by Karthik et al.^23^, the CRM^31^ framework is useful to calculate and minimize severity after the model inference. It alters the predicted labels to minimize conditional risk (*R*) as follows

7 $$\begin{aligned} \arg \min _k R({y} = k | {x}) = \arg \min _k \sum _{j=1}^K {\varvec{C}}_{k, j} \cdot p({y} = j | k) \end{aligned}$$

${\varvec{C}}$ represents the class relationship matrix where ${\varvec{C}}_{i, j}$ is the height of LCA between the labels $y_i$ and $y_j$, and is directly related to the severity defined in Eq. (3). *K* is the total number of classes in the considered classification task.

### Severity of a model

The severity of a sample in this work is considered as the LCA distance between the predicted and ground truth labels. This is denoted in Eq. (3). Accordingly the severity of a classifier is represented as the expectation of all the severity values over all the samples passed into the classifier as input. This is denoted by Eq. (9).

8 $$\begin{aligned} X_{s}^{(f)} = {\mathbb {E}}_{x_i, y_i \sim P(X, Y)}[\textsc {lca}(f(x_i), y_i)] \end{aligned}$$

Here $X_{s}^{(f)}$ denotes the severity of the classifier *f*. $x_i$ is the input data and $y_i$ is the corresponding ground truth label. *P*(*X*, *Y*) denotes the data distribution from which the pairs $(x_i, y_i)$ are sampled.

Equations (3) and (8) are useful to calculate the severity in a multiclass setting, however in the multilabel setting such a formulation does not provide the correct results as it incurs a loss of information. Thus the above framework is extended for multilabel scenario as follows: consider a predicted-label vector $\varvec{{\hat{y}}}$ and a ground truth vector ${\varvec{y}}$. Let $\varvec{{\hat{y}}}(i)$ and ${\varvec{y}}(i)$ represent the values at index *i* in predicted label vector and ground truth vector, respectively. The severity for a multilabel task setting can thus be defined as:

9 $$\begin{aligned} {\mathcal {S}} = \frac{1}{|J|}\sum _{i \in I} \sum _{j \in J} \textsc {lca}(i, j) \end{aligned}$$

Here the set $I = \{i\, | \, \varvec{{\hat{y}}}(i) = 1$ and ${\varvec{y}}(i) \ne 1\}$ and the set $J = \{j\, | \, \varvec{{y}}(j) = 1$ and $\varvec{{\hat{y}}}(j) \ne 1\}$.

A classifier gives more severe errors when the average severity of the classifier is quantitatively larger than the other. Note that CRM as formulated in Eq. (7) is not suitable for a multilabel setting due to combinatorial intractability. To make it useful for multilable setting, the CRM output obtained as ${\varvec{C}} \cdot {\varvec{p}}$, where ${\varvec{C}}$ is the class relationship matrix and ${\varvec{p}}$ is the prediction probability vector, is simply considered as the soft labels representing multilabel prediction. The classifiers are compared based on the mean severity value as denoted in Eq. (8). Each classifier is first trained on the standard binary cross entropy loss, and then its severity value is checked with and without the CRM framework. Further, the classifiers are trained on the HXELoss function, and the severity values are compared against the standard results.

## Results

### Experimental setup

The models are examined using two different severity-reducing frameworks namely the CRM framework^23^, and the HXELoss based training^28^. We evaluate seven popular classification models namely Resnet18, Resnet50 Densenet121, Wideresnet50, EfficientnetB4, MobilenetV2 and ShufflenetV2 on the Chexpert dataset. The image size is kept to $(224 \times 224)$ to limit the GPU memory usage of the training process. The models are trained for 25 epochs with a batch size of 64 using the adam optimizer with the learning rate set to 0.001. The standard loss function used for training is the binary cross entropy loss.

**Table 1:** Average severity of models trained using the BCELoss.

Model	AUROC		F1 Score		SoE	
	Standard	CRM	Standard	CRM	Standard	CRM
Resnet18	77.38 ± 0.01	75.56 ± 0.56	0.75 ± 0.01	0.73 ± 0.03	2.37 ± 3.99	8.75 ± 3.70
Resnet50	77.96 ± 0.01	76.62 ± 0.06	0.71 ± 0.01	0.71 ± 0.03	1.48 ± 2.58	10.93 ± 4.63
Densenet121	79.07 ± 0.00	77.78 ± 0.07	0.73 ± 0.01	0.71 ± 0.02	2.29 ± 2.99	10.06 ± 4.61
MobilenetV2	74.17 ± 0.01	72.78 ± 0.07	0.59 ± 0.01	0.61 ± 0.02	2.83 ± 3.36	9.42 ± 4.62
ShufflenetV2	76.29 ± 0.01	76.50 ± 0.07	0.53 ± 0.01	0.54 ± 0.02	2.03 ± 2.87	10.15 ± 4.46
Wideresnet50	78.68 ± 0.02	78.60 ± 0.06	0.55 ± 0.00	0.58 ± 0.03	2.29 ± 2.99	11.04 ± 4.55
EfficientNetB4	75.48 ± 0.01	74.45 ± 0.03	0.57 ± 0.01	0.54 ± 0.04	1.71 ± 2.69	10.63 ± 4.56

The results are compared by applying CRM as a post-inference technique for reducing the severity values. The severity of a model does not often depend upon the high AUROC scores and it can be noted that simpler models with lower AUROCs perform better as compared to more complex models.

### Preparing the dataset

The CheXpert dataset^9^ contains chest radiographs with 14 labels corresponding to different thoracic diseases. Since there is a built-in hierarchy of labels in this dataset each sample can have more than one label attached to it. For a comprehensive analysis we train the models on this dataset using two different labeling policies. The dataset is split into a train set of 166,028 samples and a validation set of 25,001 samples. All the results are on the validation set as the test set only contains 224 samples. All samples are split with respect to their patient ids so as to avoid overlaps. First one is the conventional multilabel classification in which the standard cross-entropy loss does not provide good results. Our experiments use the binary cross-entropy loss function (BCELoss).

The second policy is to covert the multilabel problem into a multiclass classification by considering only one of the positive labels (“*one*”) and assign “*zeros*” to all the others for each sample. This is a more rudimentary approach and leads to loss of information. However, this is more compatible for the loss functions like the HXELoss, which is discussed in the next subsection.

### Reductions in severity using CRM and HXELoss

Table 1 shows the severity values in the standard setting without any severity-reducing framework applied to the model. It is then compared to the models where a post-inference framework, CRM, is applied to reduce the severity values.

From Table 1, it can be observed that the CRM framework does not help in reducing the average severity of the classifiers. Instead a large increase in the severity value is observed in each model that has been tested. It is also observed that AUROC (or F1-Score) is not a factor which can be relied upon when making a prediction for the severity value as models with high AUROC and complex architectures (Densenet121, WideResnet50) perform worse than simpler models (Mobilenet, Shufflenet, Resnet18) with a low AUROC value.

**Table 2:** Average severity of models trained using the HXELoss.

Model	AUROC		F1 Score		SoE	
	BCE	HXE	BCE	HXE	BCE	HXE
Resnet18	$77.38 \pm 0.01$	$67.88 \pm 0.02$	$0.75 \pm 0.01$	$0.67 \pm 0.01$	2.37 ± 3.99	5.97 ± 3.94
Resnet50	$77.96 \pm 0.01$	$70.23 \pm 0.01$	$0.71 \pm 0.01$	$0.71 \pm 0.00$	1.48 ± 2.58	5.98 ± 3.97
Densenet121	$79.07 \pm 0.00$	$69.57 \pm 0.01$	$0.73 \pm 0.01$	$0.76 \pm 0.01$	2.29 ± 2.99	5.98 ± 4.11
MobilenetV2	$74.17 \pm 0.01$	$61.87 \pm 0.01$	$0.59 \pm 0.01$	$0.59 \pm 0.00$	2.83 ± 3.36	6.04 ± 4.12
ShufflenetV2	$76.29 \pm 0.01$	$63.60 \pm 0.00$	$0.53 \pm 0.01$	$0.55 \pm 0.00$	2.03 ± 2.87	6.38 ± 4.31
Wideresnet50	$78.68 \pm 0.02$	$64.97 \pm 0.01$	$0.55 \pm 0.00$	$0.58 \pm 0.01$	2.29 ± 2.99	6.23 ± 4.07
EfficientNetB4	$75.48 \pm 0.01$	$64.28 \pm 0.02$	$0.57 \pm 0.01$	$0.81 \pm 0.01$	1.71 ± 2.69	6.23 ± 4.11

Similar to CRM framework, HXELoss also fails to improve severity values of models.

**Figure 3:**
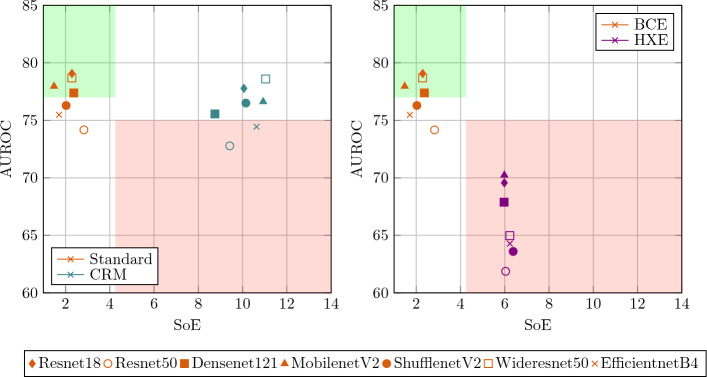
Severity vs AUROC plot in (left) standard vs CRM setting and (right) BCE vs HXE setting. The models falling in the upper left corner region of the plot have a comparatively higher AUROC and lower severity and, thus are preferable. In contrast, the models falling in the lower right corner region are comparative less desirable.

In the next experiment, where the models are trained using the HXELoss, a similar phenomenon is observed, as shown in Table 2. HXELoss is also found to be ineffective in reducing the severity value for the models. From Table 2, it can again be noted that the higher AUROC (or F1-score) does not ensure a lower severity value.

It can also be observed that HXELoss is not very efficient in training the considered models. This is due to the fact for HXE the problem is diluted from multilabel to multiclass classification (this is discussed in “Preparing the dataset”). Thus the models result in suboptimal AUROC since the loss function cannot capture the desired information about the whole dataset. However, despite a lower score, it can be noted that directly incorporating the hierarchy is more efficient in reducing the severity when compared to the previously used post-inference framework that is CRM.

## Discussion

**Figure 4:**
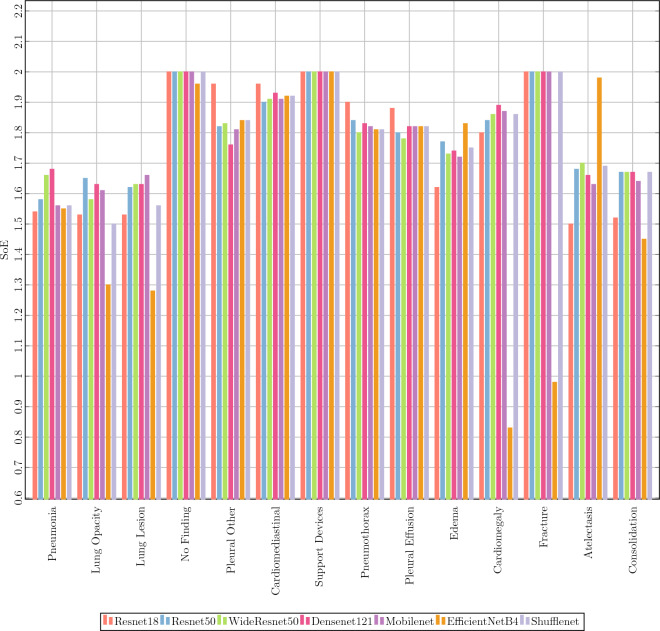
Severity for all the models for all labels. Maximum value of severity for an individual class is bounded by the height of the hierarchy tree. In case of CheXpert it is 2.

From the experiments it is observed that a high AUROC score (or F1 score) does not necessarily translate into a low SoE value for the deep learning models. Furthermore, deep and complex architecture, which often result in high AUROC, do not guarantee low SoE. This indicates that there exists a trade-off between the quantities giving more importance to the correctness of the predictions like AUROC and the others which focus on the consequences of the incorrect predictions like SoE. Therefore, when only the former is used for optimization during training, latter suffers. To understand this better consider the AUROC vs SoE plots shown in Fig. 3 for the Standard vs CRM and BCE vs HXE settings. The models falling in the upper left corner of the plot show high AUROC and low severity values. These models are more desirable compared to the other considered models for the task at hand. In the current analysis, Resnet18, Wideresnet50, Densenet121 and MobilenetV2 show the best severity to AUROC ratio and thus fall into the plot’s upper left corner region. In contrast, the models falling in the lower right corner region have low AUROC and high severity. Surprisingly Resnet50, which is one of the most popular choice of deep neural network architectures, is in lower right corner region after applying CRM and it, therefore, is a relatively less preferable model in the considered scenario. Furthermore, In the models like EfficientnetB4 (before and after the use of CRM) are relatively less suited for deployment. Figure 3 also indicates that while HXELoss reduces the severity of models, it is not able to train the models for a multilabel classification task properly and the models show low severity but also the low AUROC values.

**Figure 5:**
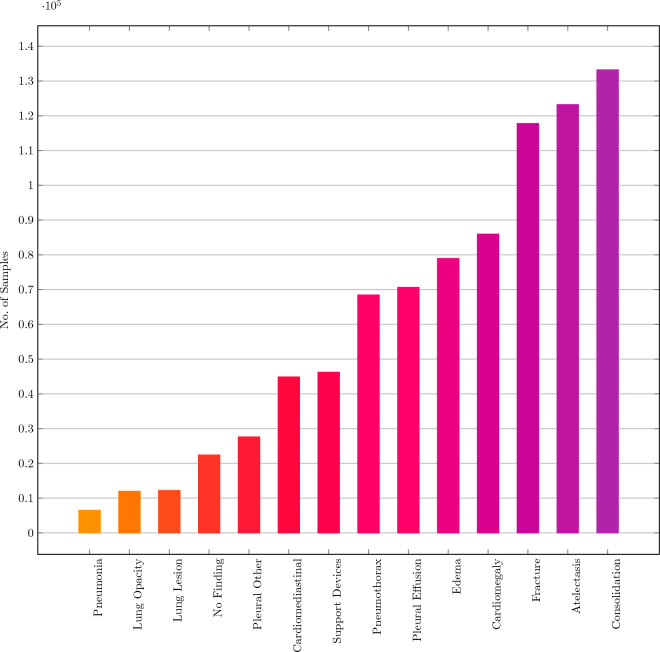
Number of samples in each class of the CheXpert dataset.

Further the results indicate that:

- In general more complex and heavier models with large number of parameters generally had more severe errors than the ones having much simpler architectures. We believe this is mainly because big models often suffer from the sample memorization problem which adversly affects their performance on the test set.
- This inverse trend is also noticed for the AUROC and the F1 Score for the models where *resnet18* performed better than *resnet50* or *wideresnet50*.
- Further an unbalanced dataset does not point to the fact that the class with less number of samples will have the highest severity. This is depicted in the manuscript’s Fig. 5.
- Using an existing as well as modified post-inference technique in multilabel classification has the inverse effect i.e. rather than decreasing the SoE it increases it further and thus it is better to incorporate SoE into the model through a training procedure.

Thus, there is a need for better frameworks and optimization criteria that incorporate class taxonomy into the model training for various different tasks in the medical domain.

To understand the above observations better, we further consider label-wise SoE values for all the considered models that have been trained using the standard BCELoss, as shown in Fig. 4. These are obtained without using any severity-reducing frameworks. It is observed that the samples belonging to the nodes at level-1 of CheXpert hierarchy tree have severity values close to 2, which is an upper bound of severity value for an individual incorrect label prediction. This is because the samples in level-1 (*Fracture, Support Devices, No Finding*) are commonly misclassified into the classes in level-2 which belong to a different branch of the hierarchy tree. Thus the only common ancestor that they have is the root. One possible reason to such misclassification is that the total number of samples corresponding to level-2 is considerably higher than that of level-1, which can be seen from Fig. 5. Reducing the severity of these samples should thus involve the prediction of samples that lie in the same branch of the hierarchy tree.

Another important observation in this work is that the frameworks which are designed to work well in multiclass classification setting fail to improve SoE in the multilabel setting. In addition frameworks like HXELoss fail in the redesigned multiclass classification setting for CheXpert dataset. This is due to the fact that these frameworks are designed for *type-1* hierarchy whereas the CheXpert has *type-2* hierarchy. Thus there is a need to look for severity reducing methods that take into account *type-2* in a better way and can move AI closer to the stage of deployability in the medical domain.

## Scope for future work

The concept of SoE is being introduced for the multi-label classification framework. The proposed approach is very general. Here we have used it for images using models designed specifically to handle feature extraction for images. However, the proposed method would work for any data type, including time series data, for a classification-based task using a suitable deep learning model provided that the data is organized in some hierarchical structure. One such example in the medical datasets would be ECG datasets^32, 33^ which can be used for classification using LSTMs. However as currently formulated it is difficult to integrate it into the training paradigm. This is due to the non-differentiable nature of the $\hbox {LCA}$ function. Thus this is an open problem for the future. Further, work needs to be done to make it an integral component of AI model development pipeline for medical applications. The future scope of the work also includes the development of dedicated severity-reducing frameworks for multi-label datasets having *type-2* hierarchies. This can be attempted through the use of quadratic weighted kappa (QWK)^34^ is a loss function that can be integrated with severity as it is designed to penalize an error that is farther from the ground truth. Vargas et al.^35–37^ explore leveraging ordinal classification of data to improve classification, and data augmentation techniques for medical and quality assessment fields. These methods require ordinal data where each label can be considered as a part of a set ${\mathcal {C}}_q$. These methods require a hierarchical decomposition of the labels according to their order. Vargas et al. use ordinal classification techniques for weapon stock quality assessment where each label is distributed into sets and an ensemble method is used to predict each label which leverages these sets that are prepared in an ordinal manner. Mondero et al. leverage a hierarchical model for the classification of Melanoma in patients to develop better decision support systems (DSS). These approaches factor in the order of a given data as they are not currently utilized in datasets that have complex hierarchies and further where each node can be a label. Further, the methods have been utilized for the standard classification task while a multi-label classification scenario has not been attempted while considering the severity of error or hierarchical datasets. Thus, ordinal classification techniques can also be seen as a useful way of introducing severity into problems where a direct hierarchy tree is not available from the data but some order can be derived due to the nature of the data and further SoE can be used to improve upon ordinal classification tasks. Thus, this is a valuable open problem that needs to be solved for the current AI models to become deployable in the medical space.

## Conclusion

This work introduces the concept of error severity for the deep learning based medical data analysis methods. Commonly used performance measures like accuracy and AUROC fail to take into account the consequences of errors made by the classifiers. However, the push towards deployable AI in healthcare can only be carried out if the model is trusted to produce minimal errors of the least associated risk. While a model may appear very accurate, it is difficult to tell if it can make errors bearing a low cost. This work infers that such apprehensions are not unreasonable as many high accuracy/AUROC models with complex frameworks produce errors of high severity that can lead to potentially dangerous consequences if taken at face value. The proposed SoE is a potential parameter that can be considered when performing a classification/labelling task. Through this work, it can also be concluded that the SoE of a model internally depends on its ability to achieve a specific performance on a label; however, it is not guaranteed that even at the same AUROC, a more complex will have a better severity value (Tables 1, 2). The severity-reducing frameworks considered here are effective in reducing the severity in a multiclass classification setting however, they are not suitable for the multilabel datasets having *type-2* hierarchies that are common in the medical domain. This work lays down the foundation for error severity of deep learning models. In particular the idea of severity is generalized from multiclass to multilabel settings.

## Data Availability

The main data (CheXpert data) used for experiments is available at https://aimi.stanford.edu/chexpert-chest-x-rays for users with credentialed access.

## Code availability

The code for the set of experiments is available in the following repository: https://github.com/Satwik-S9/Severity-of-Error-in-Hierarchical-Datasets.
